# Supplementary material for: The effects of genital myiasis on the diversity of the vaginal microbiota in female Bactrian camels
Source: BMC Vet Res. 2022 Mar 5;18:87. doi: 10.1186/s12917-022-03189-5 (PMC8897907; doi:10.1186/s12917-022-03189-5)
Supplement: Supplementary file 5 — Additional file 5. [file 12917_2022_3189_MOESM5_ESM.zip › MPL201709200_16s_yy/Treat1/css/js_css/jsPDF-master.zip!/jsPDF-master/docs/plugins_svg.js.html]

plugins/svg.js - Documentation


## Home

### Classes

- jsPDF

### Global

- addFont
- addHTML
- addMetadata
- addPage
- autoPrint
- CapJoinStyles
- circle
- ellipse
- getFontList
- lines
- lstext
- output
- rect
- roundedRect
- save
- setDisplayMode
- setDrawColor
- setFillColor
- setFont
- setFontSize
- setFontStyle
- setLineCap
- setLineJoin
- setLineWidth
- setPage
- setProperties
- setTextColor
- text
- triangle

# plugins/svg.js

```
/** @preserve
jsPDF SVG plugin
Copyright (c) 2012 Willow Systems Corporation, willow-systems.com
*/
/**
 * Permission is hereby granted, free of charge, to any person obtaining
 * a copy of this software and associated documentation files (the
 * "Software"), to deal in the Software without restriction, including
 * without limitation the rights to use, copy, modify, merge, publish,
 * distribute, sublicense, and/or sell copies of the Software, and to
 * permit persons to whom the Software is furnished to do so, subject to
 * the following conditions:
 *
 * The above copyright notice and this permission notice shall be
 * included in all copies or substantial portions of the Software.
 *
 * THE SOFTWARE IS PROVIDED "AS IS", WITHOUT WARRANTY OF ANY KIND,
 * EXPRESS OR IMPLIED, INCLUDING BUT NOT LIMITED TO THE WARRANTIES OF
 * MERCHANTABILITY, FITNESS FOR A PARTICULAR PURPOSE AND
 * NONINFRINGEMENT. IN NO EVENT SHALL THE AUTHORS OR COPYRIGHT HOLDERS BE
 * LIABLE FOR ANY CLAIM, DAMAGES OR OTHER LIABILITY, WHETHER IN AN ACTION
 * OF CONTRACT, TORT OR OTHERWISE, ARISING FROM, OUT OF OR IN CONNECTION
 * WITH THE SOFTWARE OR THE USE OR OTHER DEALINGS IN THE SOFTWARE.
 * ====================================================================
 */

;(function(jsPDFAPI) {
'use strict'

/**
Parses SVG XML and converts only some of the SVG elements into
PDF elements.

Supports:
 paths

@public
@function
@param
@returns {Type}
*/
jsPDFAPI.addSVG = function(svgtext, x, y, w, h) {
	// 'this' is _jsPDF object returned when jsPDF is inited (new jsPDF())

	var undef

	if (x === undef || y === undef) {
		throw new Error("addSVG needs values for 'x' and 'y'");
	}

    function InjectCSS(cssbody, document) {
        var styletag = document.createElement('style');
        styletag.type = 'text/css';
        if (styletag.styleSheet) {
        	// ie
            styletag.styleSheet.cssText = cssbody;
        } else {
        	// others
            styletag.appendChild(document.createTextNode(cssbody));
        }
        document.getElementsByTagName("head")[0].appendChild(styletag);
    }

	function createWorkerNode(document){

		var frameID = 'childframe' // Date.now().toString() + '_' + (Math.random() * 100).toString()
		, frame = document.createElement('iframe')

		InjectCSS(
			'.jsPDF_sillysvg_iframe {display:none;position:absolute;}'
			, document
		)

		frame.name = frameID
		frame.setAttribute("width", 0)
		frame.setAttribute("height", 0)
		frame.setAttribute("frameborder", "0")
		frame.setAttribute("scrolling", "no")
		frame.setAttribute("seamless", "seamless")
		frame.setAttribute("class", "jsPDF_sillysvg_iframe")

		document.body.appendChild(frame)

		return frame
	}

	function attachSVGToWorkerNode(svgtext, frame){
		var framedoc = ( frame.contentWindow || frame.contentDocument ).document
		framedoc.write(svgtext)
		framedoc.close()
		return framedoc.getElementsByTagName('svg')[0]
	}

	function convertPathToPDFLinesArgs(path){
		'use strict'
		// we will use 'lines' method call. it needs:
		// - starting coordinate pair
		// - array of arrays of vector shifts (2-len for line, 6 len for bezier)
		// - scale array [horizontal, vertical] ratios
		// - style (stroke, fill, both)

		var x = parseFloat(path[1])
		, y = parseFloat(path[2])
		, vectors = []
		, position = 3
		, len = path.length

		while (position < len){
			if (path[position] === 'c'){
				vectors.push([
					parseFloat(path[position + 1])
					, parseFloat(path[position + 2])
					, parseFloat(path[position + 3])
					, parseFloat(path[position + 4])
					, parseFloat(path[position + 5])
					, parseFloat(path[position + 6])
				])
				position += 7
			} else if (path[position] === 'l') {
				vectors.push([
					parseFloat(path[position + 1])
					, parseFloat(path[position + 2])
				])
				position += 3
			} else {
				position += 1
			}
		}
		return [x,y,vectors]
	}

	var workernode = createWorkerNode(document)
	, svgnode = attachSVGToWorkerNode(svgtext, workernode)
	, scale = [1,1]
	, svgw = parseFloat(svgnode.getAttribute('width'))
	, svgh = parseFloat(svgnode.getAttribute('height'))

	if (svgw && svgh) {
		// setting both w and h makes image stretch to size.
		// this may distort the image, but fits your demanded size
		if (w && h) {
			scale = [w / svgw, h / svgh]
		}
		// if only one is set, that value is set as max and SVG
		// is scaled proportionately.
		else if (w) {
			scale = [w / svgw, w / svgw]
		} else if (h) {
			scale = [h / svgh, h / svgh]
		}
	}

	var i, l, tmp
	, linesargs
	, items = svgnode.childNodes
	for (i = 0, l = items.length; i < l; i++) {
		tmp = items[i]
		if (tmp.tagName && tmp.tagName.toUpperCase() === 'PATH') {
			linesargs = convertPathToPDFLinesArgs( tmp.getAttribute("d").split(' ') )
			// path start x coordinate
			linesargs[0] = linesargs[0] * scale[0] + x // where x is upper left X of image
			// path start y coordinate
			linesargs[1] = linesargs[1] * scale[1] + y // where y is upper left Y of image
			// the rest of lines are vectors. these will adjust with scale value auto.
			this.lines.call(
				this
				, linesargs[2] // lines
				, linesargs[0] // starting x
				, linesargs[1] // starting y
				, scale
			)
		}
	}

	// clean up
	// workernode.parentNode.removeChild(workernode)

	return this
}

})(jsPDF.API);
```

  

Documentation generated by JSDoc 3.4.2 on Sun Oct 09 2016 11:08:27 GMT+0100 (BST) using the docdash theme.
